# Adult-Onset Still’s Disease With Extensive Lymphadenopathy Mimicking Lymphoproliferative Malignancy

**DOI:** 10.7759/cureus.16163

**Published:** 2021-07-04

**Authors:** Mishouri Paul, Prodip Paul, Dipon Dey, Saba Safdar, Julio Ramos

**Affiliations:** 1 Medicine, Interfaith Medical Center, New York, USA; 2 Internal Medicine, Geisinger Community Medical Center, Scranton, USA; 3 Epidemiology and Public Health, ZWH Medical Care PC, New York, USA; 4 Internal Medicine, The Wright Center for Graduate Medical Education, Scranton, USA; 5 Rheumatology, Geisinger Community Medical Center, Scranton, USA

**Keywords:** adult-onset still’s disease, lymphadenopathy, lymphoproliferative malignancy, lymphoma, polyarthritis

## Abstract

Adult-onset Still’s disease (AOSD), a rare systemic inflammatory disorder of unknown etiology, is considered in broad differential in patients with fever of unknown origin or unexplained lymphadenopathy. It is characterized by spiking fever, evanescent salmon-colored maculopapular rash, arthritis or arthralgia, and leukocytosis. Due to broad differentials and lack of any specific diagnostic tests, diagnosis of AOSD poses a great challenge. A concerned physician should have a high index of suspicion while dealing with patients presenting with clinical symptoms of this systemic disorder. We report a case of a 25-year-old African American female with the past medical history of AOSD, who presented with four weeks history of extensive cervical and axillary lymphadenopathy mimicking lymphoproliferative malignancy. Cases have been reported with the development of malignant lymphoma during the course of AOSD. Therefore, careful monitoring of patients with regular follow-up is vital as these patients may develop lymphoproliferative malignancy in the future.

## Introduction

Adult-onset Still’s disease (AOSD) is a rare multisystemic inflammatory disorder of unknown etiology, characterized by high spiking fever, polyarthralgia, evanescent salmon-colored maculopapular rash, lymphadenopathy, and hepatosplenomegaly [1,2]. Because of the genetic differences and disease heterogeneity in the studied populations, experimental data of a possible genetic association remains unclear [3]. Several infectious agents including viruses, bacteria, and parasites have been identified with AOSD [4]. AOSD has been reported to be associated with leukemia [4], breast cancer [5], esophageal cancer [6], occult papillary thyroid carcinoma [7] and malignant lymphoma [8-10], and rheumatic disease [4]. It is a diagnosis of exclusion. After the initial presentation, a thorough diagnostic evaluation should be performed to exclude malignancy, infection, and rheumatic diseases [2].

Lymphadenopathy is a prominent feature and is found in 65% of patients [11]. Moreover, the lymph node histology after excisional lymph node biopsy in AOSD may range from reactive hyperplasia to atypical paracortical hyperplasia, resembling lymphoma [12]. As malignant lymphoma is one of the most important differential diagnoses of AOSD, it is particularly important to exclude malignant lymphoma. Moreover, cases have been reported where the long-standing history of AOSD has caused stimulation of lymphoid systems to progress towards lymphoma [8-10]. Therefore, it is particularly important to monitor these patients at regular intervals.

The course of disease in AOSD can be monocyclic, polycyclic, or chronic, and prognosis depends on the course of the disease and is more favorable when systemic symptoms predominate [4]. We present a case of a 25-year-old female who presented with extensive cervical and axillary lymphadenopathy, inflammatory polyarthritis, and sore throat. After extensive workup, infectious, malignancy, and other rheumatologic diseases were ruled out and the cause of the lymphadenopathy was considered to be due to underlying AOSD.

## Case presentation

A 25-year-old African American female with PMH of AOSD was admitted to hospital with the complaints of painful swelling of neck glands for four weeks along with pain in bilateral wrist and knee joints, and sore throat. The patient noticed gradual swelling of neck glands more marked in the right submandibular region that started approximately one month ago. Neck swelling became significantly painful, and the patient developed difficulty with swallowing two days prior to admission. The patient also complained of approximately 10-pound weight loss over the past six to eight months. The patient denied any recent travel abroad, cat bite, history of TB or exposure to TB patient, cough, or hemoptysis. The patient was diagnosed with Adult-onset Still’s Disease (AOSD) two years prior. During that time, she had a lymph node biopsy which was reportedly negative for lymphoproliferative malignancy. She had been prescribed prednisone with resolution of symptoms. Since then, the patient had episodes of symptoms of AOSD several times. The patient also reported intermittent skin rash.

On physical examination, she was found to have palpable, firm, and tender bilateral cervical lymphadenopathy besides markedly tender and enlarged right submandibular lymph nodes, with the largest one measuring approximately 3.5 x 2 cm. She also had bilateral axillary lymphadenopathy with the largest one in the left axillary region measuring approximately 3 x 2.5 cm. In addition, she had mild tenderness and swelling in bilateral wrist and knee joints.

**Figure 1:**
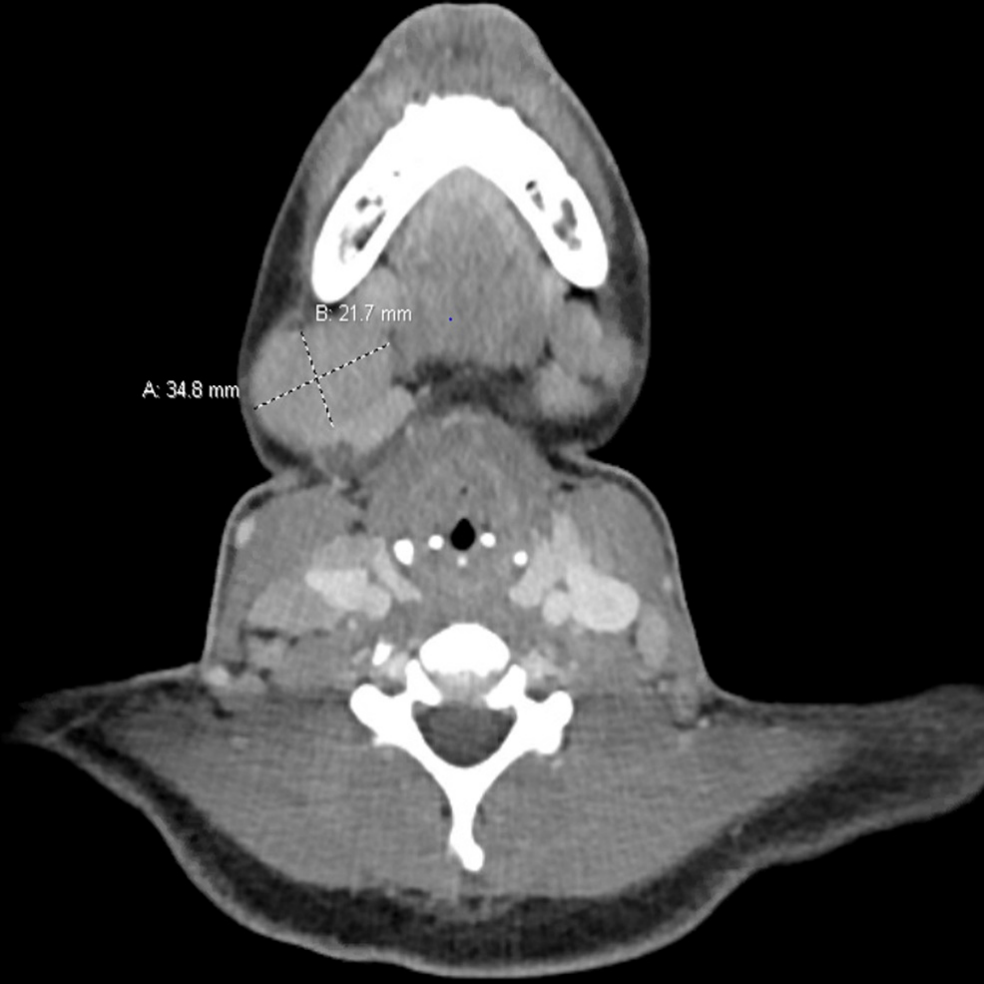
CT scan of neck with IV contrast showing bilateral cervical lymphadenopathy with the largest one measuring approximately 2.2 x 3.5 cm lymph node in the right submandibular region

**Figure 2:**
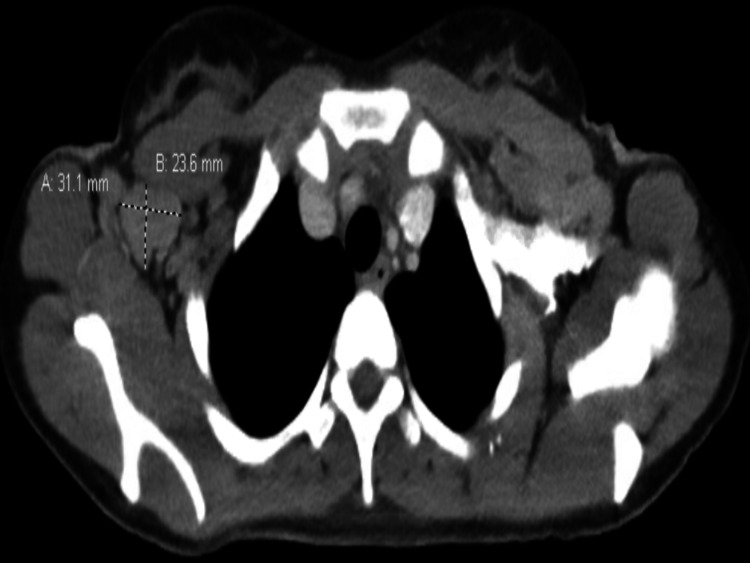
CT scan of chest with IV contrast showing bilateral axillary lymphadenopathy with the largest one measuring approximately 3.1 x 2.4 cm lymph node in the right axilla

An oncology consult was requested for evaluation of suspected lymphoproliferative malignancies. A rheumatology consult was also requested for inflammatory polyarthritis and given her history of AOSD. On admission, laboratory data showed WBC 10,610/ml, platelet count 512,000/ml, C-reactive protein (CRP) 58 mg/dl, erythrocyte sedimentation rate (ESR) 84 mm/h. Liver function tests were within normal limit. The serum ferritin level was 962 ng/ml. Specific connective tissue disease serologies were all negative including antinuclear antibody (ANA) and rheumatoid factor. Other tests that were negative included, HIV test, Lyme Ab, Epstein-Barr virus (EBV) IgM, hepatitis B and C. During the hospital stay, the patient had intermittent fever. Chest X-ray was negative for any infiltrates, CT chest of abdomen and pelvis with intravenous (IV) contrast was done for evaluation of lymphadenopathy which was also negative for any infectious focus. Urinalysis was unremarkable, urine culture, and blood cultures were negative. CT scan of neck with IV contrast showed extensive multilevel bilateral cervical lymphadenopathy with the largest one measuring approximately 2.2 x 3.5 cm in the right submandibular region (Figure 1). CT scan of chest with IV contrast showed bulky bilateral axillary lymphadenopathy with the largest lymph node measuring 3.1 x 2.4 cm in the right axilla (Figure 2). Mild hepatosplenomegaly was also noted on CT imaging.

**Figure 3:**
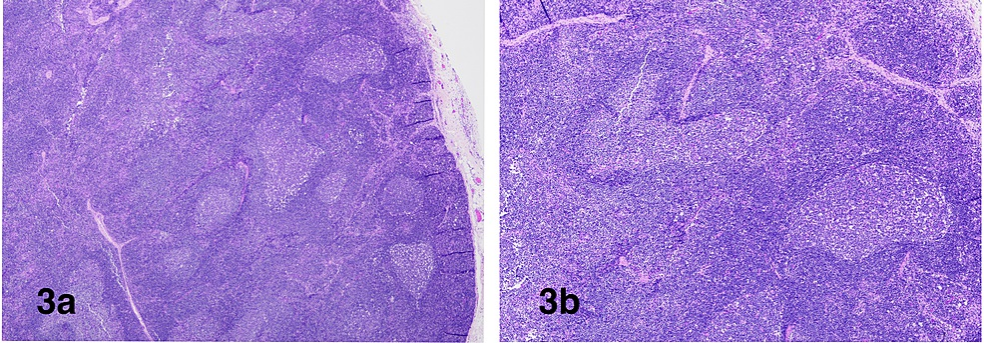
Lymph node is enlarged with reserved architecture and follicular hyperplasia. H&E stain, 20x (a) and 40x (b)

**Figure 4:**
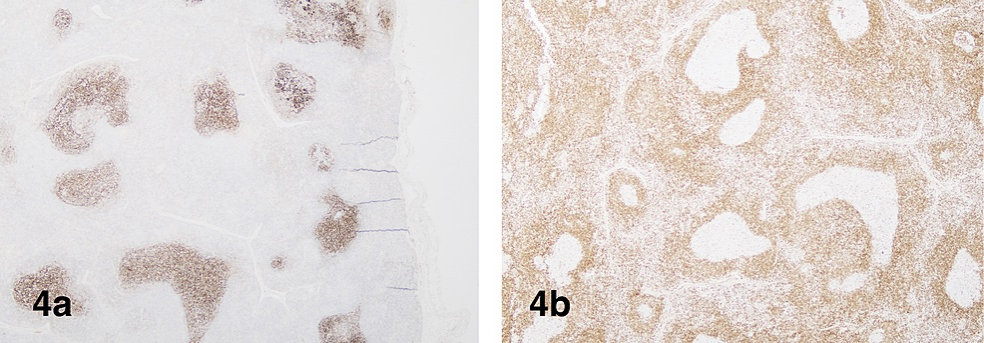
(a) CD21 shows expanded follicular dendritic cell (FDC) meshwork, 20x and (b) BCL2 is negative in the germinal centers

Excisional biopsy of cervical lymph nodes was done, which showed follicular and lymphoplasmacytic hyperplasia without definite morphological evidence of lymphoma (Figure 3a-3b). Immunohistochemical stains demonstrated expanded follicular dendritic cell (FDC) meshwork and IgG4-related disease was ruled out (Figure 4a-4b). Molecular studies for B and T-cell receptor rearrangements were negative for clonal population. Flow cytometric analysis of whole blood showed no evidence of lymphoma or acute leukemia.

Malignancies, infectious etiology, and other rheumatologic diseases were ruled out, extensive lymphadenopathy was considered to be secondary to AOSD, and the patient was started on high-dose prednisone. The patient showed marked clinical improvement after initiation of high dose steroid (60 mg/day) and was discharged after five days of hospitalization. The patient was discharged on high dose steroid with taper with outpatient Rheumatology follow-up. On follow-up visit with the Rheumatologist, the patient had marked improvement of symptoms in four weeks and clinically no appreciable palpable lymphadenopathy was noted. Repeat ferritin was 108.

## Discussion

Adult-onset Still’s disease (AOSD) is a distinct clinical entity and affects both genders equally with the majority of patients being 16-35 years of age [2]. In this case, the patient was a 25-year-old female.

Clinically, patients present with fever, rash, sore throat, and arthralgia with fever and arthralgia, being the most common manifestations. Pharyngitis is another common earlier finding in AOSD which either precedes the development of fever or can occur along with other symptoms. The characteristic rash of AOSD is a transient, nonpruritic, salmon-colored, maculopapular lesion, and is often observed during febrile episodes. AOSD most commonly involves knee, wrist, ankle, and elbow joints [4]. Rheumatoid factor and antinuclear antibody tests are generally negative and, if present, are of low titer. Corticosteroids are considered first-line treatment for management of AOSD [4]. In this case, the predominantly involved joints were bilateral wrist and knee joints. The patient had negative rheumatoid factor and antinuclear antibody test and she was started on high dose prednisone with rapid and significant improvement of symptoms.

The pathogenesis of AOSD is not completely understood. However, aberrant activation of the innate immune system with overproduction of pro-inflammatory mediators like interleukin-1, 6, 18, and tumor necrosis factor-α are thought to be associated with systemic symptoms including fever, and arthritis. These cytokines are also considered to be the cause of leukocytosis, increased acute phase reactants and ferritin levels [4]. In our patient, acute phase reactants were elevated.

It has been reported that approximately one-fifth of patients with AOSD experience long-term remission within one year, while one-third of patients may experience a complete remission followed by one or more relapses. However, the timing of relapse is unpredictable [4]. As per Fautrel, AOSD can be monocyclic, polycyclic, and chronic [3]. The polycyclic pattern of AOSD has been observed in our patient. Case reports have been published where AOSD has progressed to lymphoma. Trotta et al. reported a case of AOSD associated with an immunoblastic malignant lymphoma [8], Sono et al. reported a case that progressed to diffuse large B-cell lymphoma [9], and Otrock et al. reported a case that progressed to non-Hodgkin’s lymphoma [10] where the time elapsed from symptoms to development of lymphoma was 21, 18 and 10 months [8-10].

Lymphadenopathy is a prominent feature and is found in 65% of patients [11]. In our case, the main presenting feature was extensive bilateral cervical and axillary lymphadenopathy. Given concern regarding lymphoproliferative malignancy, lymph node biopsy was done to rule this out. Since there are reported cases of AOSD evolving to lymphoproliferative malignancy, our patient was encouraged for regular outpatient follow-up.

## Conclusions

Adult-onset Still’s disease affects primarily young adults. Due to the lack of specific clinical, laboratory, and histological features diagnosis can be incredibly challenging. Physicians should have a high index of suspicion while dealing with patients presenting with polyarthralgia, unexplained fever, and lymphadenopathy. Since AOSD has been reported to progress to lymphoproliferative malignancies, it is important to closely monitor these patients regularly.
